# Editorial: Neurotechnologies in translation: technological challenges and entrepreneurship opportunities

**DOI:** 10.3389/fnins.2023.1195756

**Published:** 2023-05-24

**Authors:** Michela Chiappalone, Alessandra Pedrocchi, Silvestro Micera, Thomas Stieglitz, Calogero Maria Oddo, Lorenzo De Michieli, Lorenzo Massa

**Affiliations:** ^1^Department of Informatics, Bioengineering, Robotics and Systems Engineering (DIBRIS), University of Genova, Genova, Italy; ^2^Rehab Technologies, Istituto Italiano di Tecnologia, Genova, Italy; ^3^Fondazione Politecnico di Milano, Milan, Italy; ^4^The BioRobotics Institute, Scuola Superiore Sant'Anna, Pisa, Italy; ^5^Translational Neural Engineering Laboratory, Center for Neuroprosthetics, Swiss Federal Institute of Technology (EPFL), Geneva, Switzerland; ^6^Department of Microsystems Engineering Biomedical Microtechnology, University of Freiburg, Freiburg, Germany; ^7^Strategy Organization and Management Group (SOM), Aalborg University Business School, Aalborg, Denmark

**Keywords:** neural disorders, neurotechnologies, business, innovation, opportunities, barriers

Nowadays neurological disorders affect more than one billion people worldwide. Given the rapid aging of the population and the related increase of diseases affecting the central nervous system, there is (and there will be) a growing demand for biomedical devices for immediate clinical use, such as neuroprostheses, exoskeletons, wearable or implantable sensors, neuromodulators, neuro-electroceutical devices, to mention some. Technological (and the related clinical) advances have the potential to turn devices such as the aforementioned ones into effective solutions to the different challenges affecting patients and also represent attractive market opportunities. For example, the market for advanced prosthetics and exoskeletons has been valued at $2.11 billion in 2018 and is expected to reach $3.82 billion by 2024, registering a Compound Annual Growth Rate of 10.42% between 2019 and 2024 (BIS Research, 2019). As of today, many different technologies are under investigation, within projects and initiatives led by both the private (e.g., companies and innovative startups) and public sectors (e.g., universities and research centers, labs, etc.). Some of these technologies and solutions have been successfully tested in controlled settings, including clinical trials, also producing important scientific achievements. Nevertheless, and despite these encouraging developments, there are still many technological, clinical as well as ethical, legal, and business barriers affecting the successful diffusion in society of devices that have the potential to provide value to those affected by neurological disorders.

Within the above depicted framework, the goal of this Research Topic is to synthesize and organize state-of-the-art insights related to different aspects of neurotechnology entrepreneurship, with a specific emphasis on the opportunities, barriers, dynamics, and mechanisms involved in translating neuro-technologies and new medical/clinical procedures from the lab to the market.

The study by Ren et al. proposes a new surgical protocol of spinal cord fusion (SCF) for paraplegic human patients. Spinal cord injury (SCI) can cause paralysis and serious chronic morbidity, and there is no effective treatment. By capitalizing on their previous experimental results of SCF in mice, rats, beagles, and monkeys, they designed a novel surgical procedure, called sural nerve transplantation (SNT), for human patients with lower thoracic SCI and distal cord dysfunction and conducted a clinical trial in 12 fully paraplegic patients. Although SNT did not restore the spinal cord continuity in white matter in some patients, SNT could restore spinal cord continuity in the cortico-trunco-reticulo-propriospinal pathway, thereby restoring in part some motor and sensory functions. They conclude that SNT may be a safe, feasible, and effective method to treat paraplegic patients with SCI. Nevertheless, additional research and clinical trials still need to be performed to optimize the procedure to be routinely used. This work shows that timing is a crucial factor in developing novel technologies and procedures as the process of testing can be long and time consuming. So, for translational research, counting on entrepreneurship capabilities and technological skills is not enough and every single step, from preclinical up to human studies, must be timely planned.

In terms of technological development, it is important to recognize that, in addition to its feasibility and human desirability, a new device should meet several criteria, such as safety, effectiveness, usability, and acceptance. With reference to prosthetic devices, embodiment is a key challenge to improve the users' acceptance and, therefore, the actual use of novel generation prosthetics. Multiple elements have been studied to improve the embodiment, initially the focus was on the natural control of the prosthetics under the direct intention of the user. In the last decade, the focus has also included the sensorial feedback to the user, proposing implanted neuroprostheses with very promising results. However, the possibility to provide tactile feedback by using transcutaneous stimulation is an interesting alternative to avoid surgery. In the paper by Rodrigues et al., a training for bidirectional control of a virtual prothesis, providing EMG control of a prosthetic knee angle by the residual leg muscles and having tactile transcutaneous stimulation feedback beyond the visual one, has been tested on seven amputees with significant improvement of a set of metrics about the embodiment. Overall, the work draws attention to the importance of incorporating the human-in-the-loop paradigm as mandatory and not ancillary in the design and the effective clinical translation of neurotechnologies.

Another important element to improve users' acceptance of a new technology is the correct identification of the end-users needs. Semprini et al. developed a user-centered-based control system for a lower limb exoskeleton named “TWIN” to provide post-stroke rehabilitation. Specifically, they describe the novel control suite named “TWIN-Acta” in terms of the conceived strategy and developmental phases and reported evaluation sessions performed on healthy clinical experts and people post-stroke to understand usability, acceptability, and barriers to usage. The system received overall good scores in terms of usability and acceptability, and it was evaluated as safe. The identified limit was related to learnability, attributable to the short-time of usage, but of course can be improved for future and prolonged training.

Advances in neuroscience and neurotechnologies raise idiosyncratic ethical, legal, and social challenges, which go beyond the boundaries of those covered by traditional bioethics. The field of neuro-ethics, the discipline that analyzes the “social, legal, ethical and policy implications of advances in neuroscience” (International Neuroethics Society, 2020), focuses on bridging this gap. The goal of the study by Moss et al. is to investigate the role of neuro-ethics vis a vis the innovation process, which is across the process that occurs when scientific discoveries, and the relative knowledge are progressively morphed into product, service and solutions which are adopted by society. The authors found that neuro-entrepreneurs' altruistic values often play an important role in their motivations to start new ventures and in shaping opportunity identification. They also found that neuro-entrepreneurs are beginning to recognize key ethical questions throughout the innovation process and forecast future ethical issues with the continued widespread use of neurotechnology. Elaborating on their findings, the authors suggest that neuro-ethics, particularly in the form of moral imagination and values, is a critical component of neuro-innovation that can even advance and enrich, instead of hinder, the neuro-industry enterprise.

Overall, the articles in this Research Topic contribute sharpening our understanding of the nature of the innovation process subtending the application and diffusion of neurotechnology. Scientific advances in neurotechnology are opening-up new opportunities for entrepreneurship. But seizing these opportunities to turn scientific discoveries into solutions that are ultimately adopted in society comes with several challenges. Entrepreneurs and scientists interested in technology transfer are confronted with the need to address technical, human (e.g., desirability, acceptability, and embodiment) and ethical challenges at the same time, just to address the proof of concept, while searching for scalable and viable business models.

To conclude, the articles in this Research Topics suggest that the design of new devices and technologies should be based, from the very beginning, on several criteria, many of which go beyond technological feasibility. Important criteria beyond those involved in technological feasibility include the needs and requirements of the end users, the ethical and legal challenges involved, and the international safety and quality standards. Furthermore, innovators are tasked with the challenge to find viable and scalable business models to create, deliver but also capture value in a sustainable way. This calls for an increasing attention to the nature of the knowledge, capabilities, skills and even mindsets that are required to successfully navigate the challenges involved in turning ideas for new devices into solutions that diffuse in society. Importantly, the skill set required lies at the intersection of different areas of knowledge and practice, including technical, human, legal, business, and even moral. We hope that this Research Topic could serve as inspiration for more work aimed at unpacking the best practices, tactics, strategies, mechanisms and skills that have the potential to support a more efficient and effective diffusion of neurotechnology.

## Author contributions

MC, LM, and AP wrote the first draft of the manuscript. All authors have made a substantial, direct, and intellectual contribution to the work and approved it for publication.
